# Comparison of the Efficacy of Intravitreal Aflibercept and Bevacizumab for Macular Edema Secondary to Branch Retinal Vein Occlusion

**DOI:** 10.1155/2016/8421940

**Published:** 2016-04-12

**Authors:** Jia-Kang Wang, Pei-Yuan Su, Yung-Ray Hsu, Yun-Ju Chen, Fang-Ting Chen, Ying-Yu Tseng

**Affiliations:** ^1^Department of Ophthalmology, Far Eastern Memorial Hospital, No. 21, Section 2, Nan-Ya South Road, Pan-Chiao District, New Taipei City 220, Taiwan; ^2^Department of Medicine, National Yang Ming University, Taipei City, Taiwan; ^3^Department of Healthcare Administration and Department of Nursing, Oriental Institute of Technology, New Taipei City 220, Taiwan; ^4^Department of Medicine, National Taiwan University, Taipei City, Taiwan; ^5^Department of Electrical Engineering, Yuan Ze University, Taiwan; ^6^Department of Medicine, Fu Jen Catholic University, New Taipei City 220, Taiwan; ^7^Department of Medicine, China Medical University, Taichung City, Taiwan

## Abstract

Fifty-two eyes of 52 patients with treatment-naïve macular edema associated with perfused branch retinal vein occlusion were retrospectively reviewed. Twenty-seven cases received PRN intravitreal bevacizumab, and 25 cases were treated by PRN intravitreal aflibercept with monthly follow-ups for 12 months. Both aflibercept and bevacizumab were effective in reduction of macular thickness and improvement of visual acuity for the participants. Both antivascular endothelial growth factor agents had similar efficacy and duration of treatment for these eyes with macular edema secondary to branch retinal vein occlusion during a 12-month period. No serious systemic or ocular adverse events were reported.

## 1. Introduction

Branch retinal vein occlusion (BRVO) is a common sight-threatening retinal vascular disorder, in which macular edema is the main cause of visual impairment [1]. Retinal ischemia after vascular occlusion can cause both vitreous and aqueous vascular endothelial growth factor (VEGF) elevation [2, 3]. Increased VEGF results in higher vascular permeability and associated macular edema in patients with BRVO. Intravitreal injections of anti-VEGF, such as bevacizumab (Avastin*™*, Genentech Inc., South San Francisco, CA, USA) and aflibercept (Eylea*™*, Regeneron Pharmaceuticals, Inc., Tarrytown, NY, USA, and Bayer Pharma AG, Berlin, Germany), can effectively lower intraocular level of VEGF and reduce vascular permeability related to macular edema in BRVO [4–14]. Herein, we performed a head-to-head comparison of efficacy after intravitreal bevacizumab and aflibercept treatments for macular edema following BRVO. To our knowledge, this is the first paper associated with the issue after reviewing the literature.

## 2. Methods

We conformed to the Declaration of Helsinki to accomplish this study. From August 2012 to September 2015, 104 eyes of 104 patients with macular edema secondary to perfused BRVO were retrospectively reviewed. The patients did not have prior trauma, intravitreal injections, retinal or macular laser, or ocular surgery except uneventful phacoemulsification. They did not have other abnormal ocular conditions. All the cases had systemic hypertension under medical treatment. No patient had history of thromboembolic events, known coagulation abnormalities or current use of anticoagulative medication other than aspirin, or other major systemic diseases. Diabetic patients with diabetic retinopathy were excluded. All the patients did not have any intravitreal anti-VEGF, macular laser, or other associated treatments. The patients with perfused BRVO were diagnosed with retinal nonperfusion area less than 10 disc diameters by fundus fluorescein angiography (TRC-NW7SF, Topcon, Tokyo, Japan). Macular edema was defined as macular leakage on fundus fluorescein angiography and central foveal thickness (CFT) more than 300 *μ*m detected by spectral-domain optical coherence tomography (RTVue, Optovue, San Francisco, USA) with macular pathologies including cystoid changes, diffuse thickening, and submacular fluid, using 6 radial line scans through the fovea in all patients. Patients with vitreomacular traction or epiretinal membrane were excluded. Baseline best-corrected visual acuity (BCVA) in Snellen chart (converting into logMAR and EDTRS letters for statistical comparison) [15], intraocular pressure via pneumotonometer (CT-80, Topcon, Tokyo, Japan), and biomicroscope of anterior segment were examined in all the patients. Once the patients were diagnosed with macular edema secondary to BRVO, intravitreal bevacizumab or aflibercept was administered within one week without accompanying macular laser or future laser rescue. The procedures were performed at Far Eastern Memorial Hospital by one surgeon (Wang JK).

After detailed explanation of risks, benefits, and off-label use of these medications, all the participants signed the informed consents before the intravitreal injections. Between August 2012 and February 2014, 54 consecutive cases received PRN intravitreal bevacizumab 1.25 mg/0.05 mL treatment. Between March 2014 and September 2015, 50 consecutive patients were treated by PRN intravitreal aflibercept 2 mg/0.05 mL. Following topical anesthesia and disinfection of eyelid and conjunctiva, aflibercept or bevacizumab was injected into the vitreous cavity using a 30-gauge needle inserted through the inferotemporal pars plana, 3.5 mm posterior to the limbus. After the procedure, tetracycline ointment was placed into the conjunctival sac. The eye was patched for one hour. The patch was removed after an hour. The patient was instructed to instill one drop of 0.3% norfloxacin into the injected eye four times daily for one week.

All the patients were followed up monthly for at least 12 months, with anterior segment and fundus examination and BCVA, CFT, and intraocular pressure measurement. The follow-up SD-OCT scans used the baseline scan as a reference. Visual testing was done in the same room at each visit. Retreatment was based on findings of optical coherence tomography including CFT more than 300 *μ*m, or there were persistent or recurrent macular cysts or submacular fluid that affected the visual acuity even if CFT is less than 300 *μ*m. Primary outcome measures included change in CFT and BCVA at month 12. Complications after injections were recorded. The intragroup changes in CFT and BCVA were compared with Wilcoxon signed rank test and the between-group numerical difference compared with Wilcoxon rank sum test. Fisher's exact test was used for categorical comparison between groups. *p* value less than 0.05 was considered significant.

## 3. Results

**Figure 1:**
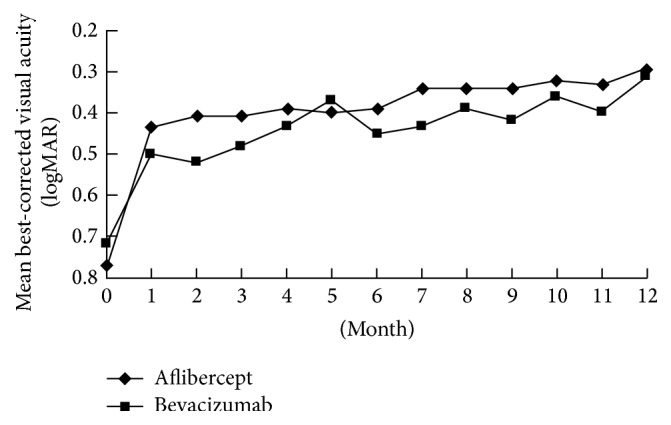
Changes of best-corrected visual acuity from baseline to month 12 in patients with macular edema secondary to branch retinal vein occlusion treated by intravitreal aflibercept or bevacizumab.

**Figure 2:**
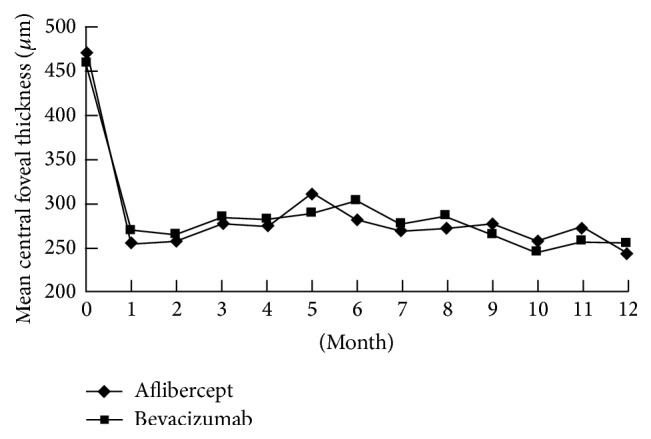
Changes of central foveal thickness from baseline to month 12 in patients with macular edema secondary to branch retinal vein occlusion treated by intravitreal aflibercept or bevacizumab.

One month after the first intravitreal aflibercept, mean BCVA significantly improved from baseline 0.77 logMAR (equal to 46.1 letters) to 0.43 logMAR (62.3 letters) (*p* = 0.0007) (Figure 1). The baseline mean CFT was 470.2 *μ*m, which significantly decreases 1 month after the first aflibercept injection to 254.2 *μ*m (*p* < 0.0001) (Figure 2). From month 2 to month 11, all time points in CFT and BCVA were significantly different from baseline data (*p* < 0.01) (Figures 1 and 2). At month 12 in aflibercept group, mean BCVA significantly improved to 0.29 logMAR (70.2 letters), significantly better than baseline BCVA (*p* < 0.0001), with mean change of −0.48 logMAR (+24.1 letters) (Figure 1). At month 12 in aflibercept group, mean CFT significantly decreased to 241.9 *μ*m (*p* < 0.0001), with mean change of −228.3 *μ*m (Figure 2).

The baseline mean BCVA was 0.72 logMAR (48.9 letters), which significantly improved 1 month after the first bevacizumab injection to 0.50 logMAR (60.1 letters) (*p* = 0.0002) (Figure 1). The mean CFT 1 month after the first intravitreal bevacizumab (268.3 *μ*m) significantly improved compared with the mean CFT at baseline (459.4 *μ*m) (*p* < 0.0001) (Figure 2). From month 2 to month 11, all time points in CFT and BCVA were significantly different from baseline data (*p* < 0.01) (Figures 1 and 2). At month 12 in bevacizumab group, mean BCVA significantly improved to 0.31 logMAR (69.5 letters), significantly better than baseline BCVA (*p* < 0.0001), with mean change of −0.41 logMAR (+21.1 letters) (Figure 1). At month 12 in bevacizumab group, mean CFT significantly decreased to 252.7 *μ*m (*p* < 0.0001), with mean change of −212.8 *μ*m (Figure 2).

There was no statistical difference between two groups in baseline data, including age, gender, lens status, incidence of patients with diabetes, presenting BCVA and CFT, and duration of symptoms (*p* > 0.05) (Table 1). At all time points from month 2 to month 11, CFT and BCVA were not significantly different between aflibercept and bevacizumab groups (*p* > 0.05). There was also no significant difference between two groups in final anatomical and functional outcome at month 12, including final BCVA, mean visual gains, final CFT, and mean CFT changes (*p* > 0.05) (Table 2). Nearly two-thirds of the patients in both groups had final BCVA equal to or more than 20/40 and visual gains more than 3 lines from baseline to month 12 (Table 2). Only less than 10% of the patients in both groups lost BCVA equal to or more than one line 12 months after the first injection (Table 2). Mean injection number of aflibercept was 2.12, comparable with that of bevacizumab (2.22) during 12-month period (*p* = 0.11). In aflibercept group, 22 (44%) eyes required no additional injection except the baseline injections. In bevacizumab group, 22 (40.7%) eyes required no additional injection except the baseline injections.

The injection was well tolerated in all patients. There were no episodes of endophthalmitis, retinal detachment, vitreous hemorrhage, or elevated intraocular pressure. The most common side effect was local hyperemia or subconjunctival hemorrhage at the site of injection. No systemic adverse events were noted.

## 4. Discussion

Macular grid laser photocoagulation was the standard of care for macular edema in perfused BRVO according to results of the Branch Vein Occlusion Study [16]. However, the visual improvement following macular laser was limited, with mean improvement of 1.33 lines of vision at the 3-year primary end point. Anti-VEGF intraocular injection has been shown to be a new promising treatment modality, which results in noticeable functional and anatomical improvement.

Bevacizumab is a full-length recombinant humanized monoclonal antibody directed against the VEGF-A. Intravitreal bevacizumab is employed to lower the intraocular VEGF level, effectively reducing macular edema in patients with BRVO [4]. The Pan American Collaborative Retina Study Group used intravitreal bevacizumab in PRN regimen to treat 63 patients with macular edema following BRVO [10]. The 2-year results demonstrated that bevacizumab 1.25 mg injection resulted in a gain of 3.8 lines. Decreased macular thickness was found, without accompanying serious ocular and systemic adverse events. Hikichi and coauthors performed PRN intravitreal bevacizumab for 89 eyes with macular edema in BRVO [5]. The 2-year results showed that injections resulted in visual gains of 0.30 logMAR and subsided macular edema. Ahn and colleagues performed PRN intravitreal bevacizumab for 69 eyes and three monthly PRN injections for 26 eyes with macular edema in BRVO [8]. The 6-month results revealed functional and anatomical improvement in both groups, and there was no significant difference between two groups in final visual acuity. Injection number was far less in PRN only regimen (mean 1.8) than in three-loading and PRN group (mean 3.4).

Intravitreal bevacizumab demonstrated superior ability than conventional macular grid laser for treating macular edema in BRVO. Russo and associates compared the efficacy of intravitreal bevacizumab and macular grid laser for patients with macular edema secondary to BRVO in a randomized study [7]. The one-year outcome demonstrated that bevacizumab injections had better visual gains and reduction of macular thickness than macular laser. Leitritz and coauthors performed a prospective crossover study to compare the efficacy of intravitreal bevacizumab and macular grid laser for patients with macular edema associated with BRVO [6]. The one-year outcome demonstrated that bevacizumab injections had superior visual improvement than macular laser. Hayashi and colleagues compared the efficacy of intravitreal bevacizumab alone and injections combined with macular grid laser for patients with macular edema in BRVO [9]. The one-year outcome demonstrated that macular laser did not have adding effect for bevacizumab injections, neither prolonging the bevacizumab effect nor bettering visual outcome in combined treatment. In this study, the patients with macular edema associated with BRVO gained 4.1 lines after one-year PRN intravitreal bevacizumab injections. The visual results were similar to prior studies, from 2.9- to 3.8-line visual improvement after one-year PRN bevacizumab treatment [5, 7, 13, 14]. There were 52.8% of patients with visual gain equal to or more than 3 lines at month 12, comparable to the outcome of previous trials (from 48% to 80%) [5, 7, 10, 13, 14]. The percentage of bevacizumab-treated patients who had at least 20/40 in BCVA was 66.6% at month 12, similar to results of prior studies after one-year PRN bevacizumab management (from 55 to 67%) [5, 10]. The mean injection number was 2.22 in one year in this study, comparable to the results of preceding articles requiring 1.9 to 2.6 injections of PRN bevacizumab within one year [5, 7, 13].

Aflibercept is a decoy receptor fusion protein, composed of the second domain of human VEGF receptor 1 and the third domain of VEGF receptor 2, which are fused to the Fc domain of human IgG1. Aflibercept can downregulate VEGF-A, VEGF-B, and placental growth factor, which are synergistic for pathologic angiogenesis. Intravitreal aflibercept was noted to lower the intraocular VEGF level in patients with neovascular age-related macular degeneration [11]. The VIBRANT study, a randomized controlled trial, was conducted in North America and Japan [12]. The study demonstrated the efficacy of intravitreal aflibercept 2 mg over the macular grid laser for 183 patients with macular edema associated with BRVO. The authors used monthly injections for six months. The 6-month results showed that the aflibercept group gained mean of 17.0 letters, significantly better than the laser group having only mean 6.9-letter improvement. The proportion of eyes that gained more than 15 letters from baseline at week 24 was 52.7%. Decrease of macular thickness was more prominent in the aflibercept group than in the laser group, without accompanying serious ocular and systemic adverse events. In this study, we had 19.2-letter visual gain in the first 6 months of PRN aflibercept injections, compared to the results of VIBRANT study which had 17-letter improvement. There were 61.1% of patients having at least 15-letter gain after 6-month aflibercept treatment, similar to 52.7% in VIBRANT study. We only used mean 1.33 aflibercept injections in the first 6 months, compared to 6 injections in the VIBRANT study.

Aflibercept revealed superior anti-VEGF capability than bevacizumab in vitro. The binding affinity of VEGF for this drug is higher than for ranibizumab and bevacizumab [17]. Aflibercept displays a prolonged VEGF inhibition in comparison with the other VEGF-antagonists ranibizumab and bevacizumab in retinal pigment epithelium/choroid organ cultures [18]. Aflibercept also demonstrates better safety than bevacizumab in vitro associated with mitochondria exocytosis [19]. In a randomized controlled trial, aflibercept was compared with bevacizumab for treatment of diabetic macular edema [20]. The authors concluded that aflibercept was more effective than bevacizumab for visual improvement in diabetic patients with poor baseline vision, but comparable efficacy in both pharmaceutical agents for better baseline vision. Both medications showed similar safety profiles and injection number during one-year follow-up.

To our knowledge, there is no publication comparing clinical outcome of aflibercept and bevacizumab for patients with macular edema secondary to BRVO. In the present study, aflibercept had comparable efficacy, injection number, and safety as bevacizumab for patients with macular edema associated with BRVO. The limitations of our study were retrospective and short follow-up period. A large prospective randomized study can further confirm the difference to treat macular edema in BRVO in longer follow-up period.

In summary, intravitreal aflibercept and bevacizumab had similar efficacy and duration of treatment for macular edema associated with BRVO during 12-month period. No serious systemic or ocular adverse events were reported.

## Figures and Tables

**Table 1:** Comparison of baseline data between intravitreal aflibercept and bevacizumab for macular edema secondary to branch retinal occlusion.

	Aflibercept (*n* = 50)	Bevacizumab (*n* = 54)	*p* value
Age (years)	63.3 ± 6.9	62.7 ± 5.5	0.34
Gender (male : female)	13 : 12	12 : 15	0.12
Lens status (phakic : pseudophakic)	22 : 3	20 : 7	0.09
Diabetic patients	10	16	0.19
Duration of symptoms (days)	22.1 ± 6.2	25.8 ± 7.4	0.23
Central foveal thickness (*μ*m)	470.2 ± 99.3	459.4 ± 96.5	0.15
Best-corrected visual acuity (logMAR)	0.77 ± 0.43	0.72 ± 0.51	0.17

**Table 2:** Comparison of clinical data after 12-month treatment of intravitreal aflibercept or bevacizumab for macular edema secondary to branch retinal occlusion.

	Aflibercept (*n* = 50)	Bevacizumab (*n* = 54)	*p* value
Final BCVA (logMAR)	0.29 ± 0.37	0.31 ± 0.34	0.28
Changes in BCVA (logMAR)	−0.48 ± 0.41	−0.41 ± 0.34	0.39
Changes in BCVA (ETDRS letters)	24.1 ± 20.1	21.1 ± 19.4	0.35
Final BCVA ≥ 20/40	36/50 (72%)	36/54 (66.6%)	0.44
BCVA gains ≥ 3 lines	30/50 (60%)	28/54 (52.8%)	0.12
BCVA loss ≥ 1 line	2/50 (4%)	4/54 (7.4%)	0.15
Final CFT (*μ*m)	241.9 ± 26.1	252.7 ± 27.8	0.26
Changes in CFT (*μ*m)	−228.3 ± 97.7	−212.8 ± 87.5	0.17
Injection number	2.12 ± 1.26	2.22 ± 1.31	0.11

BCVA: best-corrected visual acuity.

CFT: central foveal thickness.
